# Supplementary material for: Nationwide trends and regional variation in intravitreal injections and anti-VEGF agent use in Japan from 2017 to 2022: An analysis of the NDB Open Data
Source: PLoS One. 2026 May 7;21(5):e0348087. doi: 10.1371/journal.pone.0348087 (PMC13152162; doi:10.1371/journal.pone.0348087)
Supplement: Attachment — Submitted filename: Response_to_Reviewers.docx [file pone.0348087.s007.docx]

**Response to Reviewers**

PONE-D-25-67681R2

*Manuscript: Nationwide trends and regional variation in intravitreal injections and anti-VEGF agent use in Japan from 2017 to 2022: an analysis of the NDB Open Data*

# Journal Requirements

**Reference list review:** We have reviewed the reference list for completeness and accuracy. No retracted papers are cited. All references have been verified.

# Reviewer #2

## Major Comment 1

**Comment:** Pick one term: use "crude utilization rate (per 100,000)" everywhere, and save "incidence" for actual patient-level disease events. Also, when you first introduce G016, work the non-VEGF percentage into the main text.

**Response:** Thank you for this important point. We have made the following changes:

1. Standardized terminology throughout the manuscript, using "crude utilization rate (per 100,000 population)" consistently.

2. Removed all instances of "incidence." Specifically, "incidence rate ratio" was changed to "rate ratio," and "Standardized incidence ratios" was changed to "Observed-to-expected ratios."

3. In the Methods section where G016 is first introduced, we have added: "Across FYs 2017–2022, anti-VEGF agent-specific drug counts accounted for 98.6%–99.3% of all G016 injections (non–anti-VEGF injections, 0.7%–1.4% per year), indicating that G016 serves as a reliable population-level proxy for anti-VEGF injection activity."

## Major Comment 2

**Comment:** Add one clear sentence summarizing how much the max/min and CV shrink after standardization.

**Response:** We have added the following summary sentence to the Results section: "After indirect age standardization, the max/min ratio decreased from 4.3 to 3.55 in FY 2017 and from 2.8 to 2.29 in FY 2022, while the CV decreased from 0.228 to 0.220 and from 0.184 to 0.172, respectively, indicating that age structure explains only a modest portion of between-prefecture variation."

## Major Comment 3

**Comment:** Add (i) the assumed working correlation (exchangeable or independent), and (ii) a one-line comment on over- or under-dispersion diagnostics.

**Response:** We have added the following to the Statistical Analysis section: "Poisson generalized estimating equation (GEE) models were fitted with an exchangeable working correlation structure and a log(population) offset; robust (sandwich) standard errors were used to account for potential overdispersion."

## Major Comment 4

**Comment:** Spell out the contrast in one tight sentence: injections stayed steady while cataract and vitrectomy volumes dropped. Anchor this with prior NDB-based surgical declines—don't just imply it, state it directly in the Discussion.

**Response:** We have added the following explicit contrast to the Discussion: "Whereas Wada et al. reported that cataract extraction declined by 15.5% and vitrectomy by 13.2% during the first COVID-19 wave (April–May 2020 vs. 2019) using the same NDB Open Data, our data show that intravitreal injection volumes decreased by only 0.9% over the same period."

## Major Comment 5

**Comment:** Add a mini table summarizing FY2022 market share: Aflibercept 71.2%, Ranibizumab 18.1%, Faricimab 6.4%, Brolucizumab 4.3%.

**Response:** These market share figures are already presented in Table 1 and stated explicitly in the Results text: "In FY 2022, the shares were aflibercept 71.2%, ranibizumab 18.1%, brolucizumab 4.3%, and faricimab 6.4%." We believe this presentation is clear and avoids redundancy.

## Major Comment 6

**Comment:** Make sure the limitation that NDB aggregates by billing location, not residence, appears once in the first paragraph of Limitations.

**Response:** This limitation appears as the second sentence in the Limitations section: "Second, aggregation is based on the location of medical institutions rather than patients' residences, so cross-prefectural referrals and visits to high-volume tertiary centers may have influenced estimates of regional variation."

## Major Comment 7

**Comment:** Before resubmission, make sure the DOI works, scripts actually reproduce all tables/figures, and the README specifies NDB release numbers and access dates.

**Response:** We have verified that the Figshare DOI (10.6084/m9.figshare.31223410) is active and publicly accessible. The repository contains all derived data tables, analysis scripts, and a README file specifying NDB Open Data release numbers (4th–9th) and access dates (23 November 2025).

## Minor Comments

**Language and residual artifacts:** We have carefully proofread the manuscript to remove all tracked change artifacts.

**Comment:** Update the Fig 1 caption to match your new wording—it still says "anti VEGF injections," but the y-axis shows anti VEGF agents plus a solid line.

**Response:** We confirm that the y-axis label ("The number of intravitreal anti-VEGF injections") and the caption ("the national total number of intravitreal anti-VEGF injections") are consistent. The bars represent drug-specific counts for each anti-VEGF agent, and the solid line indicates the sum of all four agents.

**Figures:** For Figure 2 (monthly patterns), we have added a note in the Figure 2 and figure legend: “Golden Week” and "The dip in May corresponds to Golden Week, Japan's consecutive national holidays."

**Friedman reporting:** χ² and degrees of freedom are reported. Kendall's W = 0.97 is retained as the effect size.

**House style:** P values are standardized as p < 0.001 where applicable. Hyphenation and thousands separators are consistent throughout.

# Reviewer #3

## Comment 1

**Comment:** Results of the impact of the COVID-19 were not mentioned.

**Response:** The COVID-19 findings are included in the Abstract: "Injection volumes showed no marked decline during the pandemic; April–May 2020 totals (119,100 injections) were only 0.9% lower than the same months in 2019 (120,202 injections)."

## Comment 2

**Comment:** Although the results of crude rate are shown in Table 2, it is better to show the age-standardized value in the main text.

**Response:** The age-standardized findings are presented in the Results section: "In FY 2017, the max/min ratio decreased from 4.3 (crude) to 3.55 (indirect), and the CV decreased from 0.228 to 0.220. In FY 2022, the corresponding values were 2.8 to 2.29 and 0.184 to 0.172." Full details remain in Supplementary Tables S3–S4.

## Comment 3

**Comment:** The analysis method might not be appropriate. The current analysis simply compared the values of the three periods, and the results indicated that the time trend effect exists. This is not an effect of the COVID-19 effect. It is better to change the analysis method or the title of the analysis.

Response: We agree with the reviewer. We have changed the section title from "Impact of the COVID-19 pandemic" to "Temporal patterns before, during, and after the COVID-19 pandemic" in both the Methods and Results sections. We have also revised the Discussion to acknowledge that our analysis cannot isolate a causal pandemic effect because we lack a counterfactual scenario, and that the observed increases primarily reflect the underlying secular trend. To provide context, we now cite Wada et al., who reported that cataract (−15.5%) and vitrectomy (−13.2%) volumes declined sharply during the same period using the same NDB data, whereas intravitreal injections decreased by only 0.9%.

We thank both reviewers for their constructive feedback, which has substantially improved the manuscript.
