# Supplementary material for: Nationwide trends and regional variation in intravitreal injections and anti-VEGF agent use in Japan from 2017 to 2022: An analysis of the NDB Open Data
Source: PLoS One. 2026 May 7;21(5):e0348087. doi: 10.1371/journal.pone.0348087 (PMC13152162; doi:10.1371/journal.pone.0348087)
Supplement: Attachment — Submitted filename: Response to Reviewers.docx [file pone.0348087.s006.docx]

**Response to Reviewers**

Manuscript ID: PONE-D-25-67681
Title: Nationwide trends and regional variation in intravitreal injections and anti-VEGF agent use in Japan from 2017 to 2022: an analysis of the NDB Open Data
Journal: PLOS ONE

We thank the Academic Editor and reviewers for their constructive comments. We have revised the manuscript accordingly. **Line numbers below refer to the revised with Track Changes manuscript**.

**Summary of major revisions**

- Clarified that **procedure code G016** captures intravitreal injection procedures and may include **non–anti-VEGF drugs** (e.g., corticosteroids), and added **Table 1** reporting annual G016 totals alongside agent-specific anti-VEGF counts and the proportion not captured by anti-VEGF drug codes.
- Added **age-structure sensitivity analyses**: ≥75-year denominator rates and **indirect age-standardization** using two age groups (<75, ≥75), with results in Supplementary Tables S2–S4.
- Strengthened statistical reporting by emphasizing **effect sizes and 95% CIs**, adding a **Poisson GEE model** for prefecture-level repeated measures, reporting **Friedman χ²/df**, and standardizing p-value reporting.
- Expanded clinical interpretation of COVID-19 findings, noting the **time-sensitive** nature of anti-VEGF therapy and prioritization to prevent **irreversible visual loss**.
- Added clinical context for drug-market dynamics, including **faricimab uptake**.
- Removed map figures and replaced geographic displays with **tables/supplementary materials** to comply with CC BY requirements.

**Responses to Journal Requirements**

**1. PLOS ONE style requirements / file naming**

We revised the manuscript formatting and file naming to conform with PLOS ONE requirements.

**2. Funding text in Acknowledgments**

We removed funding-related text from the manuscript. “The authors received no specific funding for this work.” include cover letter.

**3. Data repository (acceptable public repository)**

We updated the Data Availability Statement to indicate deposition of the minimal processed datasets, intermediate tables, and analysis scripts in figshare, and we will provide the DOI/URL in the submission system (Lines **605-614**).

**4. Copyrighted map images**

We removed the map figures and replaced them with tabular summaries and supplementary tables (Results section; Supplementary Tables S2–S4).

**Reviewer #1**

**Comment 1**

A comparison to world trends would contextualize the trends reported here.

**Response:** We agree and added a brief international comparison in the Discussion, noting that several countries reported short-term declines in intravitreal injection visits during the first pandemic wave, whereas our national analysis showed minimal short-term reduction and continued growth overall.
**Changes made:** Discussion, Lines **438-442** and **471-478**.

**Reviewer #2**

**Comment 2.1**

G016 code includes intravitreal steroids; the manuscript should not treat G016 as anti-VEGF only.

**Response:** We clarified throughout that G016 captures intravitreal injection procedures irrespective of drug class and may include intravitreal corticosteroids. To address potential non-anti-VEGF “contamination,” we added a national-level sensitivity comparison by separating agent-specific anti-VEGF injections using drug codes and reporting the proportion of G016 not captured by these anti-VEGF codes (non-VEGF%). We also expanded the Discussion to clarify interpretive limits of prefecture-level crude G016 rates and to acknowledge that time- or region-specific changes in non-VEGF use cannot be fully evaluated in the Open Data.
**Changes made:** Results Lines **257-263**; **Table 1** and its legend; Discussion Lines **513-521**.

| **FY** | **G016 Total** | **Anti-VEGF Sum** | **Difference** | **Non-VEGF %** |
| --- | --- | --- | --- | --- |
| 2017 | 600,068 | 591,469 | 8,599 | 1.4% |
| 2018 | 671,768 | 663,342 | 8,426 | 1.3% |
| 2019 | 742,913 | 735,060 | 7,853 | 1.1% |
| 2020 | 789,476 | 781,965 | 7,511 | 1.0% |
| 2021 | 853,396 | 846,525 | 6,871 | 0.8% |
| 2022 | 936,715 | 929,704 | 7,011 | 0.7% |

**Comment 2.2**

Faricimab uptake and agent market dynamics; brolucizumab safety concerns should be discussed.

**Response:** We added clinical context describing likely reasons for rapid faricimab uptake (durability/dual-pathway mechanism supporting treat-and-extend) and slower brolucizumab uptake (safety concerns, including intraocular inflammation/retinal vasculitis) following its introduction. *"Faricimab achieved 6.4% market share within its first year of approval (FY 2022), indicating rapid adoption. This may reflect its bispecific mechanism targeting both VEGF-A and angiopoietin-2 (Ang-2), and the potential for extended treatment intervals of up to 16 weeks demonstrated in pivotal trials, which align well with treat-and-extend protocols widely used in Japan for neovascular AMD and polypoidal choroidal vasculopathy. In contrast, brolucizumab uptake remained modest (4.3% by FY 2022) despite approval in 2020, likely reflecting clinician caution following post-marketing reports of intraocular inflammation and retinal vasculitis."*

**Changes made:** Discussion Lines **471-478**.

**Comment 2.3**

COVID-19 “resilience” needs clinical context (time-sensitive care and risk of irreversible visual loss).

**Response:** We revised the Discussion to explicitly state that intravitreal anti-VEGF therapy is time-sensitive and that delaying treatment may cause irreversible visual loss, which likely contributed to prioritization of injections during the state of emergency. We also added the absolute April–May counts to contextualize the 0.9% change.
**Changes made:** Discussion Lines **541-546**.

**Comment 2.4**

Regional analysis and specialist distribution; referral/centrality bias; location of billing vs residence.

**Response:** We removed the specialist-density analysis (due to interpretability concerns with aggregated prefecture-level data). We expanded the limitations to clarify that prefecture-level counts reflect the location of medical institutions submitting claims, not patient residence, and that referral patterns and cross-prefecture care may influence observed regional variation.
**Changes made:** Specialist-density analysis removed; Limitations Lines **579-580**.

**Comment 2.5**

Seasonal trends (fiscal year and Golden Week) should be discussed.

**Response:** We added an explanation that the late-fiscal-year rise and the dip around late April–early May are consistent with the Japanese fiscal calendar and Golden Week holidays, which can affect clinic schedules and appointment timing.
**Changes made:** Discussion Lines **438–442**.

**Comment 2.6**

Statistical analysis and reporting (effect sizes/CI; count-data models; multiple comparisons; p-value formatting).

**Response:** To keep changes limited while addressing this concern, we (i) reported slopes and 95% confidence intervals for annual trends; (ii) added a Poisson GEE model with a log(population) offset for repeated prefecture-level observations, reporting IRRs with 95% CIs for regional indicators and their time interactions; (iii) reported Friedman χ² statistics and degrees of freedom; and (iv) standardized p-value reporting (e.g., p < 0.001 where applicable). Regarding multiplicity, we clarified that analyses were primarily descriptive/exploratory; therefore, we did not apply formal multiple-testing adjustments, and we interpret p-values cautiously while emphasizing effect sizes and confidence intervals.
**Changes made:** Statistical analysis Lines **238-242**.

**Comment 2.7**

Age standardization is needed for time and regional comparisons.

**Response:** We added two sensitivity approaches addressing age structure: (i) recalculation of rates using the ≥75-year population as the denominator (FY2017 and FY2022), and (ii) indirect age-standardization using two age groups (<75, ≥75) to compute SIRs and indirectly standardized rates. These results are provided in Supplementary Tables S2–S4 and summarized in the Results.
**Changes made:** Methods Lines **153–162**; Results Lines **354–377**; Supplementary Tables **S2–S4**.

**Comment 2.8**

Reproducibility and data transparency (share scripts/derived tables; specify NDB release).

**Response:** We specified the NDB Open Data releases used (4th–9th releases, corresponding to FY2017–FY2022) and updated the Data Availability Statement to provide the processed datasets, intermediate tables, and analysis scripts in figshare to enable full reproduction.
**Changes made:** Methods Lines **110**.

**Minor comments**

• Abstract: The statement “0.9% lower in April–May 2020 vs 2019” lacks context and leaves readers guessing. Include the actual numbers or expected volumes for those months so it’s clear whether 0.9% is meaningful or negligible.
**Response:** April–May 2020 totals (119,100 injections) were 0.9% lower than the same months in 2019 (120,202 injections). We added specific figures.

• Introduction: The opening paragraphs get lost discussing general AMD/VEGF benefits. Trim the generic background and focus right away on Japan’s unique context—high PCV prevalence, an aging population, and the novelty of using the NDB. The intro feels weak right now. Spell out the specific knowledge gap from the start.
**Response:** We couldn't reduce it much because we need to cite the literature later.

• Lines 143–145 (Methods): The tense jumps between past and present when describing data sources. Keep it consistent.
**Response:** We revised.

• Methods: You say you ran Friedman tests for seasonality, but only report “p<0.001.” Include the test statistic and degrees of freedom—something like “χ²(df)=X, p=Y”—so readers can see the full results.
**Response:** We revised.

• Figure legends: Define any abbreviations the first time they appear in captions (e.g., FY, NDB). Spell out “FY2022” as “fiscal year 2022,” etc., for clarity.
**Response:** This figure has been deleted. We have also checked the other figures.

• Figure 4: Change the Y-axis label to “Injections per 100,000 population.” Mention “FY2022” in the title or axis label. Add small tick marks or annotations for fiscal year end and Golden Week on the time-series plots so readers can quickly spot those dips and spikes.
**Response:** This figure has been deleted.

• Maps (Figs. 2–3): Add the class intervals or color legend details (quantiles, etc.) so the shading thresholds are obvious.
**Response:** This map has been deleted.

• Table 1: Add confidence intervals for the annual mean rates, not just mean±SD. Mean±SD only describes the data; CIs show how precise those estimates are.
**Response:**

• Drug Market Share: Instead of hiding the agent percentages in the text, use a simple table or bullet list:
FY2022: Aflibercept 71.2%, Ranibizumab 18.1%, Faricimab 6.4%, Brolucizumab 4.3%.
This format also sets up a discussion about why Faricimab’s share climbed so fast.
**Response:**

• Abbreviations: Spell out “PCV” (polypoidal choroidal vasculopathy) the first time you mention it, especially since it helps explain aflibercept’s dominance in Japan.
**Response:**

• Hyphenation/Spelling: Make sure “anti-VEGF” is written the same way throughout (some spots have an en-dash). “Board-certified ophthalmologists” needs a hyphen. Also, check for inconsistent spacing around parentheses and symbols.
**Response:** We also checked the other parts.

• “Statistical” typo: I noticed “statisitical” somewhere—run a global search and fix it.
**Response:** We revised.

• Number formatting: Pick one style for thousands (e.g., “1 093.0” with a space or comma) and stick with it throughout to avoid confusion.
**Response:** We also checked the other parts.

• Discussion: The paper ends abruptly without any global perspective. Add at least one sentence comparing Japan’s trends to those in countries like South Korea or Australia (claims-based studies are out there) to put the findings in an international context. As a reviewer put it, “Who ends a discussion without mentioning Korea?!”
**Response:** 34.	Kim J-G, Kim YC, Kang KT. Impact of Delayed Intravitreal Anti-Vascular Endothelial Growth Factor (VEGF) Therapy Due to the Coronavirus Disease Pandemic on the Prognosis of Patients with Neovascular Age-Related Macular Degeneration. J Clin Med. 2022;11: 2321. doi:10.3390/jcm11092321　Added new references.

• Data Policy statement: Specify whether you used the 7th or 8th NDB Open Data release. This detail helps others reproduce your work.
**Response:** We specified the NDB Open Data releases used (4th–9th releases, corresponding to FY2017–FY2022) and updated the Data Availability Statement to provide the processed datasets, intermediate tables, and analysis scripts in figshare to enable full reproduction.
**Changes made:** Methods Lines **110**.

• English usage: The manuscript is mostly clear, but it needs a careful grammar and typo check. Examples: don’t write “p < 0.001 (0.000)”—just use “p < 0.001.” Avoid redundant phrases like “very very significant”—one “very” is enough. Double-check subject–verb agreement.
**Response:** p < 0.001 was standardized.

• Order of Discussion: I agree with another reviewer—discussing policy and regulation in the middle of the discussion feels awkward. Cover limitations first, then finish with broader implications for a smoother flow.

**Response:** The discussion section has been revised.

We believe these revisions have substantially strengthened the manuscript. We thank the reviewers again for their thorough and constructive feedback.
